# Kentucky State Medical Society

**Published:** 1851-11

**Authors:** 


KENTUCKY STATE MEDICAL SOCIETY.

The readers of the Journal will perceive that a State Medical Society was formed recently at Frankfort, and as it deserves the confidence and good will of the profession, we hope it will command those elements of success. B.

